# Raman Optical Activity of 1T-TaS_2_

**DOI:** 10.1021/acs.nanolett.1c04990

**Published:** 2022-04-03

**Authors:** Ewa M. Lacinska, Magdalena Furman, Johannes Binder, Iaroslav Lutsyk, Pawel J. Kowalczyk, Roman Stepniewski, Andrzej Wysmolek

**Affiliations:** † Faculty of Physics, University of Warsaw, Pasteura 5, 02-093 Warsaw, Poland; ‡ Faculty of Physics and Applied Informatics, University of Lodz, Pomorska 149/153, 90-236 Lodz, Poland

**Keywords:** 1T-TaS_2_, charge density waves, Raman optical activity, resonant effects, polarization-resolved experiments

## Abstract

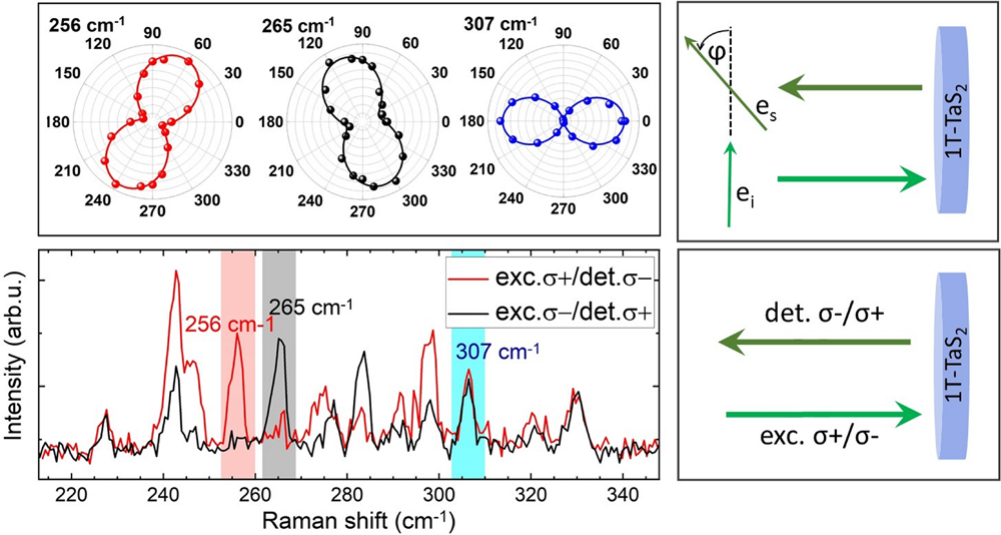

Measurements of optical activity can be readily performed in transparent matter by means of a rotation of transmitted light polarization. In the case of opaque bulk materials, such measurements cannot be performed, making it difficult to assess possible chiral properties. In this work, we present full angular polarization dependencies of the Raman modes of bulk 1T-TaS_2_, which has recently been suggested to have chiral properties after pulsed laser excitation. We found that a mechanical rotation of the sample does not alter polarization-resolved Raman spectra, which can only be explained by introducing an antisymmetric Raman tensor, frequently used to describe Raman optical activity (ROA). Raman spectra obtained under circularly polarized excitation demonstrate that 1T-TaS_2_ indeed shows ROA, providing strong evidence that 1T-TaS_2_ is chiral under the used conditions of laser excitation. Our results suggest that ROA may be used as a universal tool to study chiral properties of quantum materials.

## Introduction

Since the first observation of optical activity of quartz by Arago in 1811,^1^ the measurement of rotation of polarization of transmitted light is one of the fundamental methods to study the chirality of materials. Such measurements can be readily performed in the case of transparent materials but cannot be applied to bulk metals because no light is transmitted. This fact makes it difficult to optically assess the chiral properties of complex systems like the charge density wave (CDW) compounds 1T-TaS_2_ or 1T-TiSe_2_.^2^ In this work, we provide evidence for the optical activity of 1T-TaS_2_ by using polarization-resolved Raman spectroscopy.

1T-TaS_2_ undergoes temperature-dependent phase transitions accompanied by periodic lattice distortions related to charge density waves.^3−5^ It adopts the following four CDW phases: an undistorted (metallic), incommensurate CDW (ICCDW), nearly commensurate CDW (NCCDW), and completely commensurate CDW (CCDW). The electronic and surface structure of 1T-TaS_2_ in different phases has been extensively studied by angle-resolved photoemission spectroscopy (ARPES) and scanning tunneling microscopy (STM) techniques.^6−9^

Despite these phase transitions, 1T-TaS_2_ can persist in mixed-metastable states^10,11^ or can be used as reversible memristor material.^12,13^ An intriguing property of 1T-TaS_2_ is the presence of a hidden state, which can be enabled by an ultrafast light pulse^14,15^ and which can also influence the CDW stacking in the out-of-plane direction.^16^ A recent work shows that changes in the CDW stacking can even be caused by incoherent white light illumination.^17^ These findings highlight that light illumination can directly influence the CDW properties of 1T-TaS_2_, and currently it is not established how results obtained by optical spectroscopy are affected by these effects. Our research demonstrates that Raman scattering in bulk 1T-TaS_2_ is a complex phenomenon, which must be analyzed in terms of the influence of light illumination, resonant effects, and optical activity.

## Experiment

Bulk 1T-TaS_2_ (hqGraphene) crystals were examined by means of Raman spectroscopy using a Horiba T64000 spectrometer. Low-temperature measurements (5 K) were performed using a liquid helium flow cryostat (Oxford Instrument Hires2). Lasers with wavelengths of 442, 532, 633, and 785 nm were used as an excitation source. The laser beam was focused by a 50× objective to an ∼1 μm spot size, and the power of ∼300 μW was chosen to avoid heating effects and damage of the sample.

For linear polarization-resolved Raman experiments, incident light polarization was fixed. A half-wave plate was mounted in front of the CCD camera, followed by a linear polarizer, which was set parallel to the incident light polarization axis (see Supporting Information). In order to collect spectra dependent on the sample rotation, a half-wave plate was mounted above the cryostat, providing conditions corresponding to a sample rotation experiment (see Supporting Information).

Circularly polarized incident light was obtained by placing a quarter-wave plate behind the first polarizer, allowing for excitation by right- (σ+) or left-handed (σ−) polarized light. A second quarter-wave plate was placed in front of the second polarizer before the CCD camera (see Supporting Information). This setup allowed for collecting Raman spectra in four different configurations of excitation/detection: σ+/σ+, σ+/σ–, σ–/σ+ and σ–/σ–.

## Results

**Figure 1:**
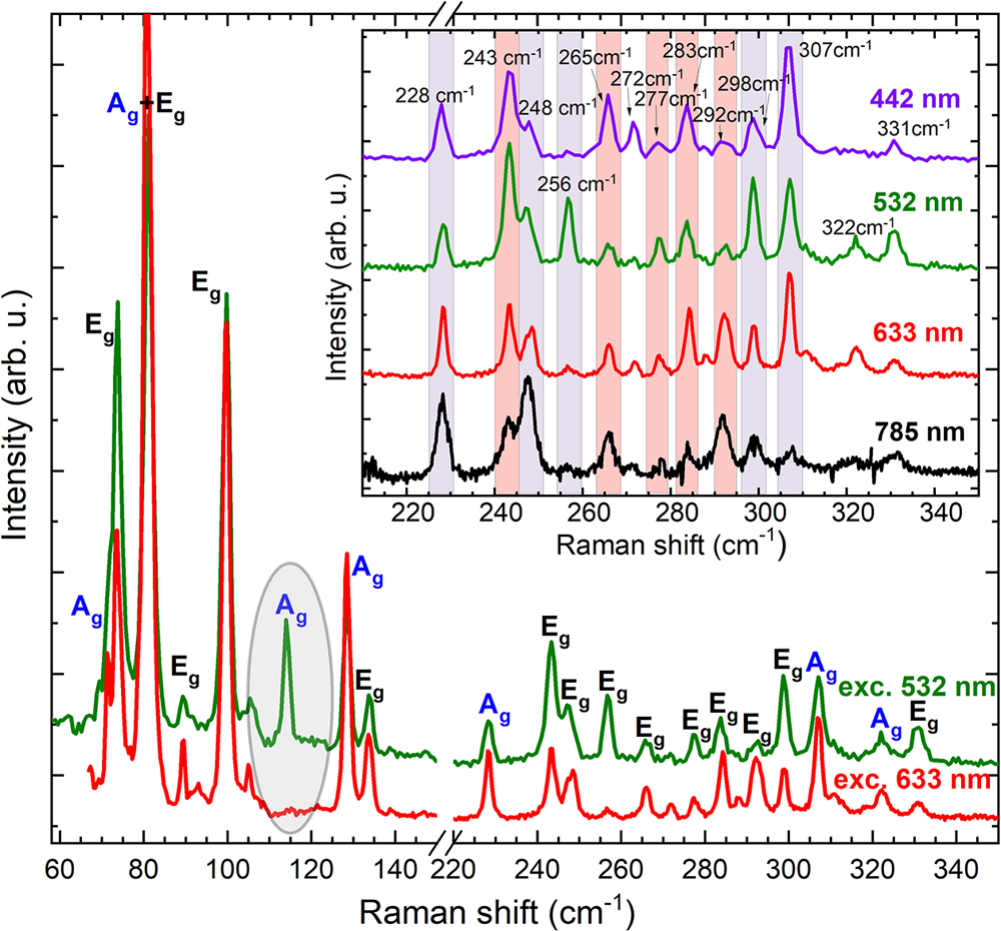
Unpolarized Raman scattering spectra of bulk 1T-TaS_2_ excited with 532 nm (green line) and 633 nm (red line) laser line. The A_g_ or E_g_ symmetry is assigned to each mode, based on polarization-resolved measurements (see Figures 2 and 3). The 114 cm^–1^ line, which is completely attenuated when excited with 633 nm wavelength laser, is marked with a gray oval. The inset shows spectra excited with different lasers: 442, 532, 633, and 785 nm wavelength. Orange and gray shadows mark modes with low (<0.5) and high (>0.5) polarization degree, respectively (under 532 nm wavelength excitation). All spectra were measured at 5 K.

Unpolarized Raman scattering spectra of bulk 1T-TaS_2_ measured with different excitation wavelength (442, 532, 633, and 785 nm) are shown in Figure 1. All spectra were measured at 5 K, that is, in the CCDW phase. Under 532 nm laser excitation, 20 Raman modes with energies close to 72, 74, 81, 89, 100, 114, 128, 134, 228, 243, 248, 256, 265, 277, 283, 292, 298, 307, 322, and 331 cm^–1^ were detected. It is commonly accepted that the observed phonon bands originate from folding of the Brillouin zone as 1T-TaS_2_ undergoes CCDW phase transition.^18−20^ As can be seen, for different excitation energies, some lines do not significantly change their intensities (228 cm^–1^), however, most of them show observable differences. A notable example is the line at 114 cm^–1^ that is completely attenuated for 633 nm excitation. The doublet with lines at 243 and 248 cm^–1^ changes its intensity ratio. Under higher (lower) excitation energy the 243 cm^–1^ (248 cm^–1^) line is more pronounced. The lines at 256 and 298 cm^–1^ are most intense under 532 nm excitation, whereas the 292 cm^–1^ line is more pronounced for lower energy excitation (633 and 785 nm wavelengths). The lines at 256, 283, and 307 cm^–1^ are very weak when excited with a 785 nm wavelength laser. Table 1 contains the intensity percentage of Raman modes, which is defined as the percentage of intensity of each line to the integrated intensity of all bands in the spectral range of 200–340 cm^–1^, excited with 442, 532, 633, and 785 nm wavelength lasers.

**Table 1:** 1T-TaS_2_ Raman Modes Intensity Percentage for Spectra in the 200–340 cm^–1^ Energy Range Excited with 442, 532, 633, and 785 nm Wavelength Lasers

	intensity percentage (%)			
Raman mode energy (cm^–1^)	442 nm exc.	532 nm exc.	633 nm exc.	785 nm exc.
228	9.4	5.7	8.9	15.4
243	19.6	18.2	11.3	11.1
248	6.2	8.9	9.8	22.9
256	1.4	8.6	1.3	1.6
265	11.5	3.5	5.3	10.3
271	5.1	0.7	1.7	2.7
277	2.3	3.2	2.8	1.5
283	10.1	7.0	9.6	7.9
292	4.7	3.1	13.9	4.9
299	7.0	11.0	6.7	4.7
307	20.7	14.9	15.5	6.2
322	<0.1	8.0	8.9	6.2
330	2.0	7.2	4.2	4.6

Changes in Raman mode intensities under different excitation energies shown in Figure 1 can be explained in terms of resonant Raman scattering, which was reported for other TMDC materials.^21,22^ When the excitation energy is tuned to a maximum in the material’s joint density of states, a resonant Raman term must be taken into account, resulting in an enhancement or attenuation of a particular mode. For a better understanding of resonant effects, one must refer to the full quantum Raman scattering model,^21,23,24^ where the Raman scattering intensity depends on the excitation energy.

1T-TaS_2_ in the CCDW phase has a large number of bands in the Brillouin zone for the energy range covered by the excitation energies applied in our experiments (1.6–2.8 eV).^7,25,26^ These energies are well above the reported values for band gaps of about 100–400 meV.^7,27,28^ By increasing the energy of the excitation laser, one covers many subsequent energy states, which are located close to each other. This results in the observed effects, that is, attenuation, enhancement, increase or decrease, of the Raman intensity of particular modes, as reported for other TMDCs.^21,22^ Additionally by selecting energy states with different symmetries, the polarization properties of Raman modes can also be altered, which will be discussed later.

**Figure 2:**
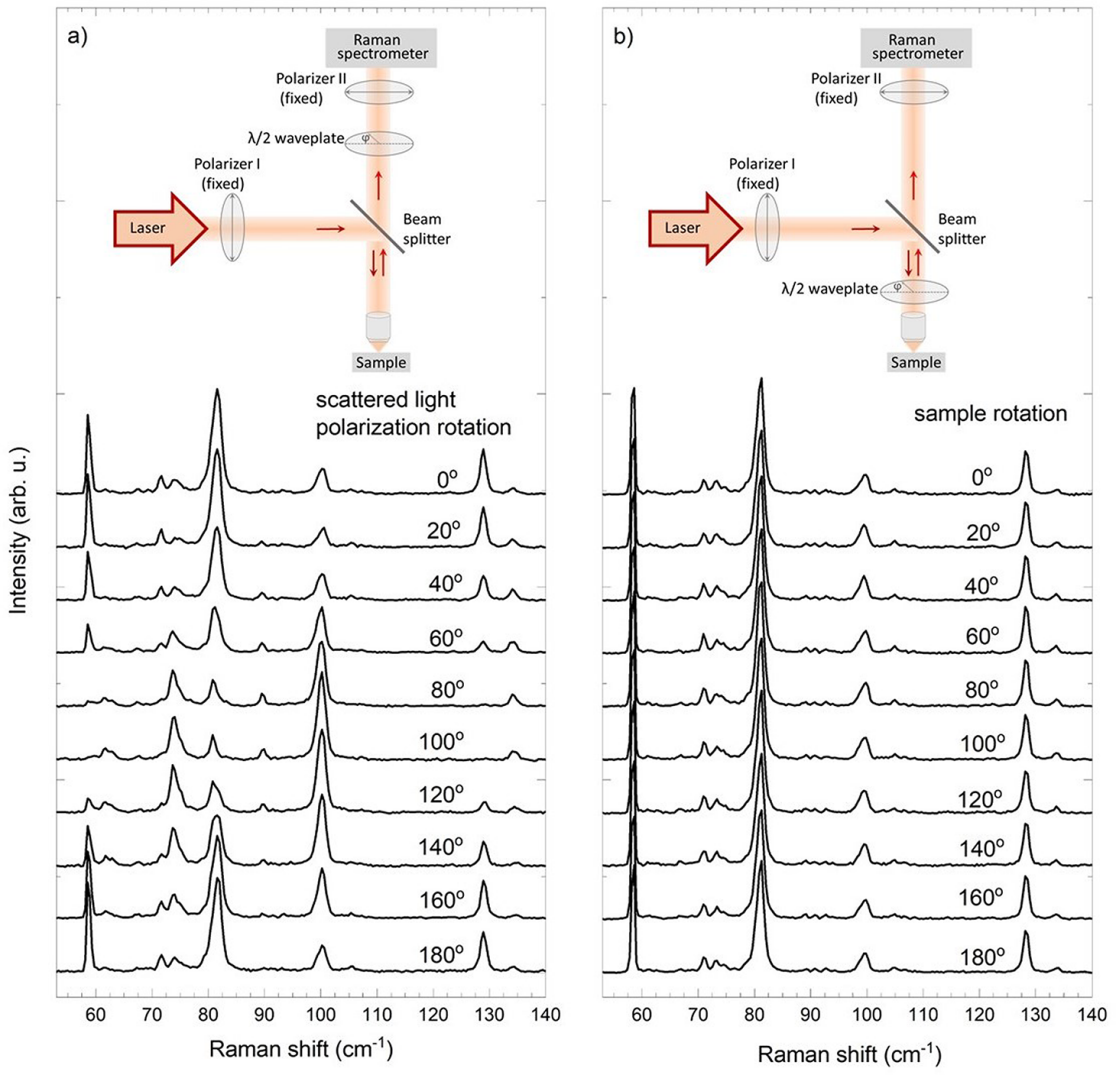
(a) Scattered light polarization rotation dependent and (b) sample rotation dependent Raman spectra of bulk 1T-TaS_2_. All spectra were measured at 5 K, excited with a 633 nm laser line. Only lower energy modes are shown for clarity. Insets show schematic drawings of experimental setups used to obtain the Raman spectra.

In order to further study resonant effects and the symmetry of Raman modes we performed polarized Raman measurements both in scattered light polarization rotation and in sample rotation configuration. The obtained Raman spectra are shown in Figure 2. As can be seen, the spectra measured as a function of the scattered light polarization show large differences (Figure 2a). All Raman modes are linearly polarized and show a maximum in intensity for different polarization angles. Raman scattering spectra measured in sample rotation configuration are shown in Figure 2b. Unexpectedly, they are entirely insusceptible to the rotation of the sample.

**Figure 3:**
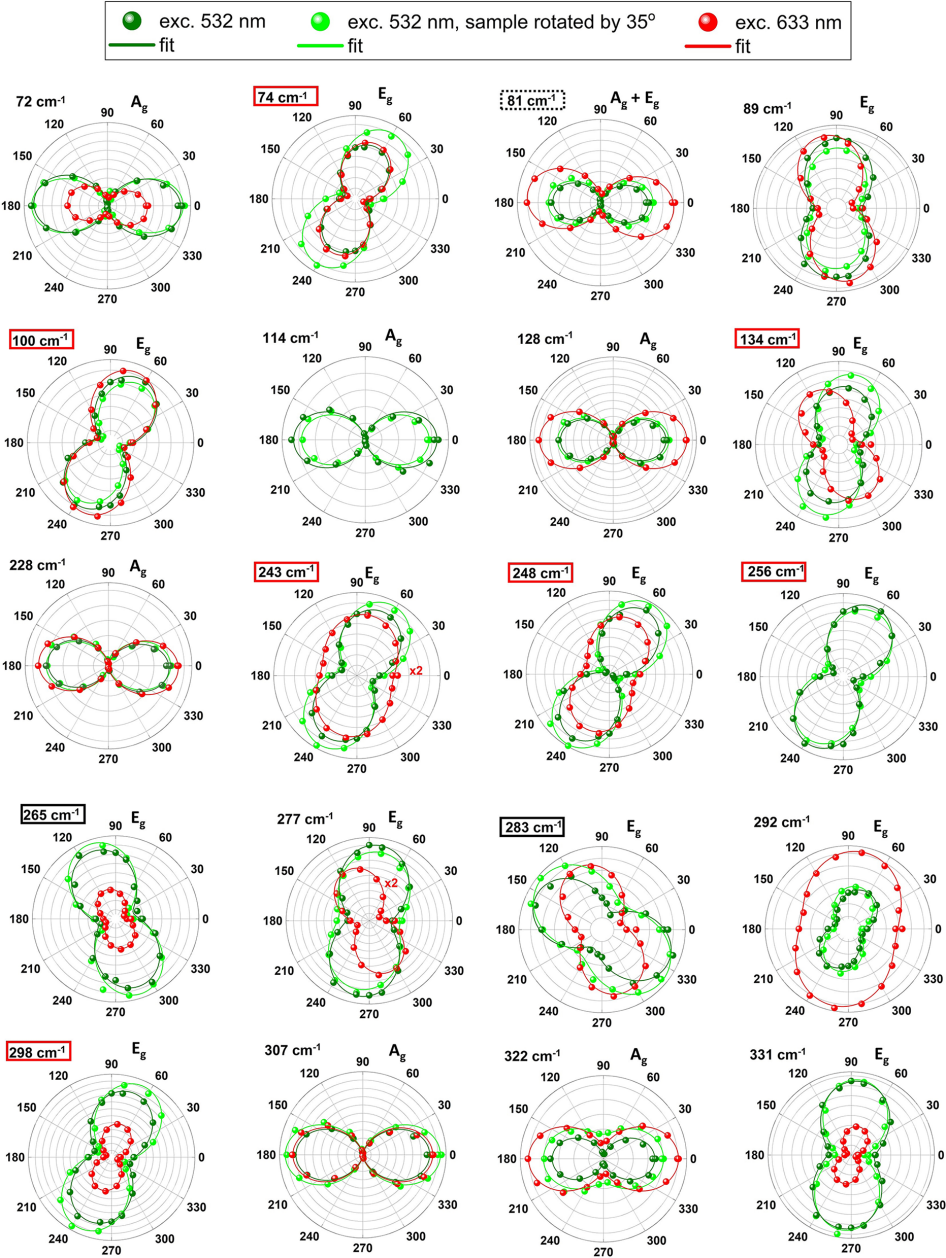
Angular intensity plots for each Raman mode measured in scattered light polarization rotation configuration with fitted function: *I* = *A* + *B* cos^2^(θ – θ_max_). Dark green circles refer to spectra excited with a 532 nm laser line. Bright green circles were measured with with 532 nm excitation but the sample was mechanically rotated by 35°. Red circles denote spectra measured under 633 nm excitation. Red and black frames mark modes more intense under σ+/σ– and σ–/σ+ configuration, respectively (see Figure 4).

Angular intensity plots obtained as a function of the scattered light polarization angle (with fixed incident light polarization) using different excitation sources (532 and 633 nm wavelength lasers) are shown in Figure 3 (*T* = 5 K). Green (red) circles show data obtained for 532 nm (633 nm) wavelength excitation. The angle θ_max_ for which the plot is the most intense was obtained by fitting the function: *I* = *A* + *B* cos^2^(θ – θ_max_), shown as green and red solid lines in Figure 3. On the basis of measurement statistics, we obtained a 5° uncertainty for the extracted θ_max_. There are lines that preserve the main axis of polarization of the laser (0°) and lines with the main axis rotated by ∼60°, ∼120° or ∼90°. Almost all lines polarized in 0° direction are fully polarized, whereas the polarization degree of lines showing other polarization angles differs from ∼0.5 to ∼0.7. Interestingly, the 248 cm^–1^ line is fully polarized when excited with green light, but weakly polarized, when excited with red light. Similarly, the 243 and 292 cm^–1^ lines are less polarized when excited with a 633 nm wavelength laser. Moreover, the polarization angles θ_max_ depend strongly on the excitation energy for a number of lines (134, 277, 283, and 292 cm^–1^). For both 134 and 277 cm^–1^ lines, the main axis changes significantly (by about 40°).

These results most probably stem from resonant effects. By changing the excitation energy, we can select states with different symmetries and therefore create resonant conditions with different symmetries, as shown in refs (22, 23, and 29). As a result, the polarization properties of Raman modes can be altered, which explains the observed main axis rotations. Resonant effects also most probably explain why the observed Raman lines are not fully polarized. When we excite 1T-TaS_2_ in the visible range, we resonantly excite many different electronic states at the same time. These states may have different symmetry properties and, consequently, angular plots consist of a mixture of different polarization directions, which results in a lower polarization degree. However, strongly polarized lines generally do not change their main axis angle, especially lines polarized in 0° direction (see Supporting Information).

According to ref (19), bulk 1T-TaS_2_ in the CCDW phase belongs to the *C*_3*i*_ symmetry point group and has two types of Raman active modes: E_g_ and A_g_. On the basis of the semiclassical model, the Raman mode intensity *I* can be written as^30^
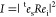
1where *e*_i_ and *e*_s_ stand for incident and scattered light electric field unit vectors, respectively, and *R* is Raman tensor for a given mode. Superscript t of *e*_s_ denotes the transformation from a column vector to a row vector. Applying appropriate Raman tensors and electric field unit vectors allows us to model the Raman intensity dependency on the scattered light polarization rotation and sample rotation (see Supporting Information). According to this theory, all lines should be linearly polarized. In the case of scattered light polarization rotation, A_g_ modes should preserve incident light polarization regardless of the sample rotation, whereas the main axis of the E_g_ modes should depend strongly on the crystal alignment with respect to the incident light polarization. This means that A_g_ modes should be polarized along 0° and should be attenuated in cross-polarization configuration, while E_g_ modes should be detectable both in co- and cross-polarization configuration. Assuming a *C*_3*i*_ crystal point group, we assigned A_g_ and E_g_ symmetry to all detected Raman modes, as shown in Figure 3. The assignment of the symmetry of A_g_ and E_g_ modes agrees well with available literature,^19,31^ where co- and cross-polarization experiments were presented.

For the sample rotation experiment, the intensity of A_g_ modes should be constant, while the angular plots of the E_g_ modes should show a four-lobbed shape. Surprisingly, angular plots obtained by mechanically rotating the sample in the cryostat by 35° are almost unaltered for both, A_g_ and E_g_ modes, as shown in Figure 3 (compare light and dark green dots). This rotation, moreover, led to a shift of the laser position on the sample, which did not affect the polarization properties, indicating the macroscopic nature of the effect. These observations cannot be explained in terms of the discussed above semiclassical approach. This unexpected result was also confirmed by rotating the linear polarization of the excitation laser on the sample. As shown in Figure 2b, all Raman modes are insusceptible to the sample rotation in respect to the incident light polarization. Interestingly, this insusceptibility to the sample rotation resembles results shown in ref (32) by Huang et al., where the angular polarization plot of the Raman mode of the two-dimensional magnet CrI_3_ A_1g_ is always rotated by the same angle with respect to the excitation laser polarization. This polarization rotation can be inverted by switching the ferromagnetic state. This behavior has not been observed in case of Raman scattering from other TMDCs in which polarization properties were analyzed based on Raman tensors and group theory.^22,23,29^ Importantly, assuming point group *D*_3*d*_ instead of *C*_3*i*_, as suggested in ref (31), also does not explain the insusceptibility of Raman modes to the sample rotation. We propose that 1T-TaS_2_ phase is optically active in the CCDW phase in agreement with a recent work showing intertwined chiral charge orders in 1T-TaS_2_ in the hidden, light-induced state.^33^

**Figure 4:**
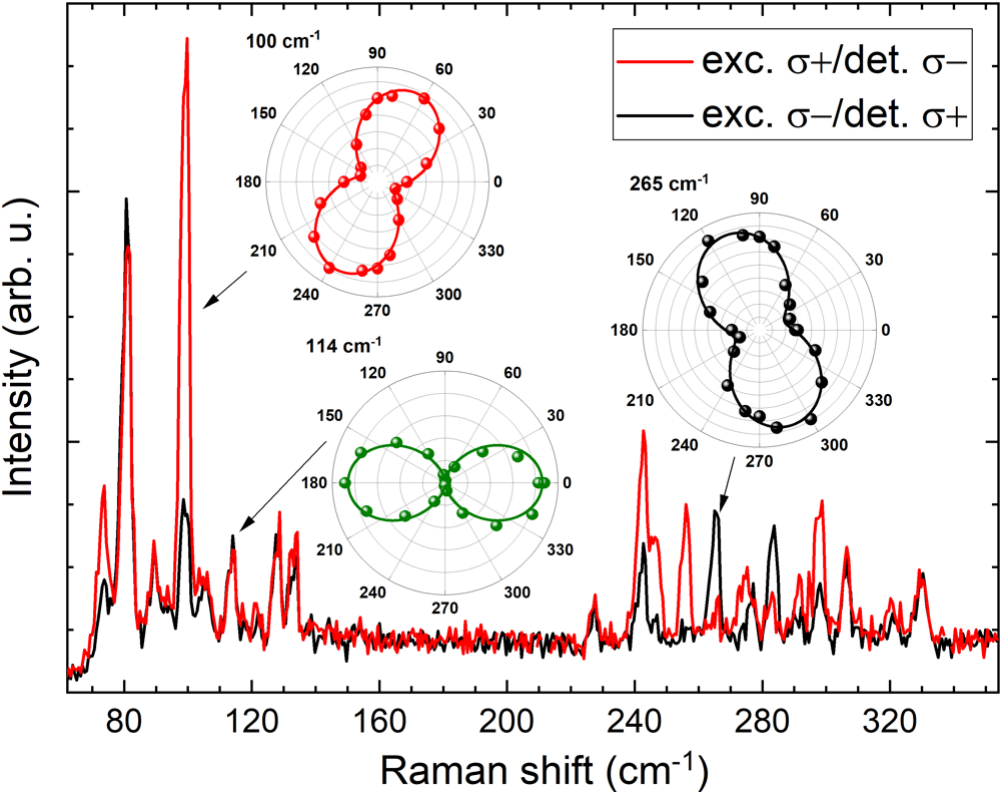
Raman scattering spectra of bulk 1T-TaS_2_ obtained in circularly polarized light in σ+/σ– and σ–/σ+ configurations, where σ+ (σ−) means right-handed (left-handed) circularly polarized light, measured at 5 K. The insets show angular plots of linear polarization of three lines, 100, 114, and 265 cm^–1^, taken from Figure 3 for comparison.

Figure 4 shows Raman spectra of bulk 1T-TaS_2_ measured in circularly polarized light in σ+/σ– and σ–/σ+ (excitation/detection) configurations, where σ+ (σ−) means right-handed (left-handed) circularly polarized light (for other configurations, see Supporting Information). As can be seen, the intensity of some lines show considerable differences, which is the signature of Raman optical activity.^34^ The lines at 74, 100, 134, 243, 248, and 298 cm^–1^ are more intense in σ+/σ– configuration, while the lines at 265 and 283 cm^–1^ are more pronounced in σ–/σ+ configuration. When we compare this data to the linear polarization angular plots (see Figure 3, green dots), we observe that all modes more intense in the σ+/σ– configuration are lines with their main axis rotated by 60–80° (modes rotated right). The only two modes with a main polarization axis of 120–150° (rotated left) are the 265 and 284 cm^–1^ lines, which clearly are more intense in σ–/σ+ configuration. Some lines remain almost unchanged in circularly polarized light. These lines are modes with a main axis of 0° or very weak modes (89, 277, and 292 cm^–1^) with a main axis close to 90°, which can be easily influenced by more intense neighboring lines, and it is not clear whether their angular plot is rotated to the left or right.

## Discussion

The observed polarization rotations are well reproduced with an antisymmetric Raman tensor in the form
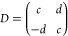
2where we omit the components related to third direction, which is not studied in our experiments. The observed polarization rotations cannot be explained in terms of chiral phonons^35,36^ found in semiconducting TMDCs (like MoS_2_) because they require the inversion symmetry to be broken, which is not the case.

Antisymmetric Raman tensors were experimentally observed by Koningstein,^37^ for yttrium aluminum garnet and introduced in eq 2 to describe Raman lines of a single crystal of PrCl_3_^38^ and by Barron^34,39^ to explain ROA for molecular light scattering. For the Raman tensor in eq 2, the Raman mode intensity according to eq 1 can be written as
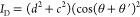
3where
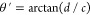
4which means that the scattered light is linearly polarized and the polarization angle is equal to θ = −θ′ and does not depend on the sample orientation. Such effects are induced by a broken mirror symmetry, which can be caused for example by the Jahn–Teller effect.^40^ It is worth noticing that the antisymmetric Raman effects induced by “chirality density” were also observed by Raman scattering for the heavy Fermion superconductor URu_2_Si_2_.^41^ In contrast to 1T-TiSe_2_, chirality has not been observed for pristine 1T-TaS_2_.^42,43^ However, recent reports show that chiral CDWs can be induced in 1T-TaS_2_ by Ti doping^43^ and in the case of the light-induced hidden state.^33^ Our results showing an insusceptibility of the polarization properties upon sample rotation and the observed Raman optical activity indicate that the studied sample is chiral. In accordance with recent results showing that the CDW stacking in 1T-TaS_2_ can be influenced even by low-power incoherent white light illumination,^17^ we believe that in fact with Raman spectroscopy we do not probe a pure CCDW state, but a light-induced “hidden” state. As shown by Gerasimenko et al.^33^ intertwined chiral charge orders can arise in this state, which may give rise to the observed optical activity. Another explanation involves the experimental backscattering configuration. Because of this configuration, only phonons with specific momentum direction are prefered and detected. Our room-temperature polarization-resolved measurements (see Supporting Information) show that Raman modes visible in the NCCDW phase can be linearly polarized with the polarization direction neither parallel nor perpendicular to the incident light polarization direction. This behavior implies that the observed optical activity is related to the superlattice formation and that some signs of it can be detected in the NCCDW phase.

**Figure 5:**
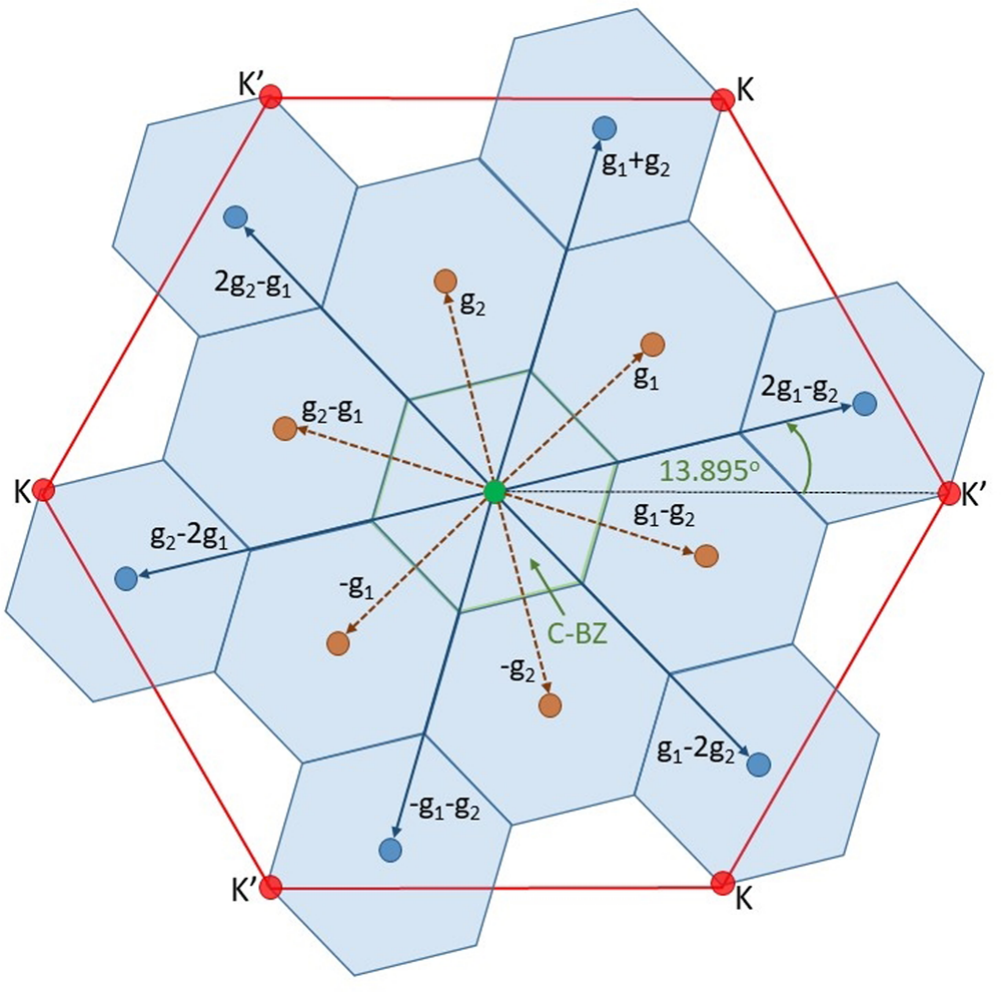
1T-TaS_2_ Brillouin zone reconstruction in the CCDW phase showing three families of points with *k*_eff_ = 0 (green, brown, and blue circles). The red hexagon shows the Brillouin zone before the metal–insulator phase transition (in undistorted phase).

A question that remains to be answered is why we observe three types of Raman modes? One type has a main axis of 0° and two more types are rotated left and right, respectively. One possible explanation may be given by the superlattice reconstruction. For the undistorted lattice there is one Γ point of the first Brilloiun zone with an effective wavevector *k*_eff_ = *k*_0_ ± *G* = 0, where *G* is a reciprocal lattice vector. However, due to the Brillouin zone reduction in the CCDW phase, we obtain two additional families of Γ points: the second group includes *k*_0_^′^ = {*g*_1_, *g*_2_, −*g*_1_, −*g*_2_, *g*_2_ – *g*_1_, *g*_1_ – *g*_2_} and the third group includes *k*_0_^″^ = {*g*_1_ + *g*_2_, 2*g*_1_ – *g*_2_, *g*_1_ – 2*g*_2_, – *g*_1_ – *g*_2_, *g*_2_ – 2*g*_1_, 2*g*_2_ – *g*_1_}. A corresponding schematic drawing is presented in Figure 5. Therefore, there are three types of Brillouin zone Γ points in the CCDW state, which can be responsible for the three types of linear polarization directions of Raman modes.

## Conclusion

We show that polarization-resolved Raman spectroscopy allows one to study complex phenomena, especially in the case of opaque bulk materials. Polarization-resolved Raman spectroscopy measurements of bulk 1T-TaS_2_ showed that the angles of the main axis of linearly polarized Raman modes cannot be explained in terms of semiclassical models. We propose that they can be understood in terms of optical activity. Our results obtained with circularly polarized light indeed show the expected Raman optical activity, which requires 1T-TaS_2_ in the CCDW phase to be chiral. The observed chirality can be explained based on recent findings showing that a chiral CDW order may be present in the light-induced state of 1T-TaS_2_. This explanation suggests that care must be taken when using laser-based methods to study CDW compounds, since light illumination may have an impact on the final properties. The polarization-resolved results are repeatable and do not depend on the position of the excitation spot on the sample, which proves that the observed effects are macroscopic crystal properties and cannot be attributed to local microscopic defects. Our results show that full polarization-resolved Raman spectroscopy, in contrast to the commonly performed cross/co linear polarization measurements, is a simple yet very powerful technique that allows studying the chirality of complex opaque materials on a macroscopic scale.

## Supporting Information Available

Experimental setup schemes; room temperature measurements; Raman tensors and angular dependencies; measurements in circularly polarized light (PDF)
