# Supplementary material for: Raman Optical Activity of 1T-TaS2
Source: Nano Lett. 2022 Apr 3;22(7):2835–42. doi: 10.1021/acs.nanolett.1c04990 (PMC9011401; doi:10.1021/acs.nanolett.1c04990)
Supplement: Supplementary file 1 — nl1c04990_si_001.pdf [file nl1c04990_si_001.pdf]

Raman optical activity of 1T-TaS_2

Ewa M. Lacinska,[*,†] Magdalena Furman,[†] Johannes Binder,[†] Iaroslav Lutsyk,[‡] Pawel J. Kowalczyk,[‡] Roman Stepniewski,[†] and Andrzej Wysmolek[†]

†*Faculty of Physics, University of Warsaw, Pasteura 5, 02-093 Warsaw, Poland*

‡*Faculty of Physics and Applied Informatics, University of Lodz, Pomorska 149/153, 90-236 Lodz, Poland*

E-mail: Ewa.Lacinska@fuw.edu.pl

Supplementary Information

Experimental setups

Schematic drawings of experimental setups used to obtain polarization-dependent Raman spectra are shown in Figs S1-S2. The setup used in sample rotation measurements was tested on a silicon sample, as shown in Fig S3.

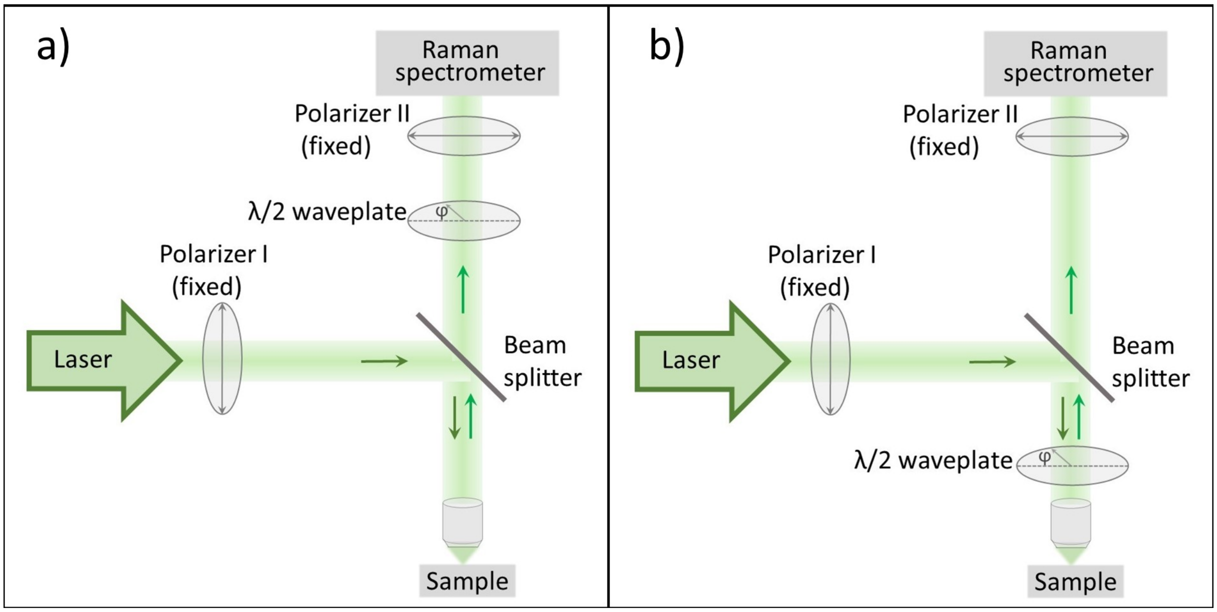

Figure S1: a) Schematic drawing of the experimental setup used to collect scattered light polarization dependent spectra. Polarizers I and II are set parallel to each other. Rotating the half-wave plate by $\theta/2$ allowed for the detection of the polarization of scattered light rotated by θ in respect to the incident laser light polarization, which means that rotating the half-wave plate from 0° to 180° in respect to the polarizers' main axis allowed for detection of Raman spectra depending on the scattered light polarization angles from 0° to 360°. Co- and cross-polarization configuration correspond to the alignment of the half-wave plate at 0° (θ_{co} =0°) and 45° (θ_{cross} =90°), respectively. b) Schematic drawing of the experimental setup used to collect Raman spectra corresponding to the sample rotation experiment. The setup yielded the expected results in case of silicon crystal, as shown in Fig. S3.

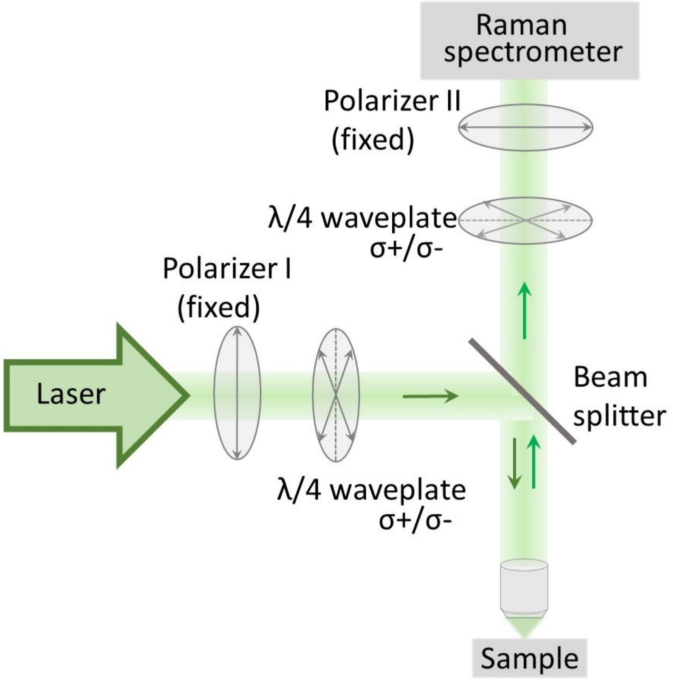

Figure S2: Schematic drawing of the experimental setup used to collect Raman spectra measured in circularly polarized light (incident and scattered). The quarter-wave plate is set with its fast axis rotated by ±45° in regard to the laser's linear polarization axis.

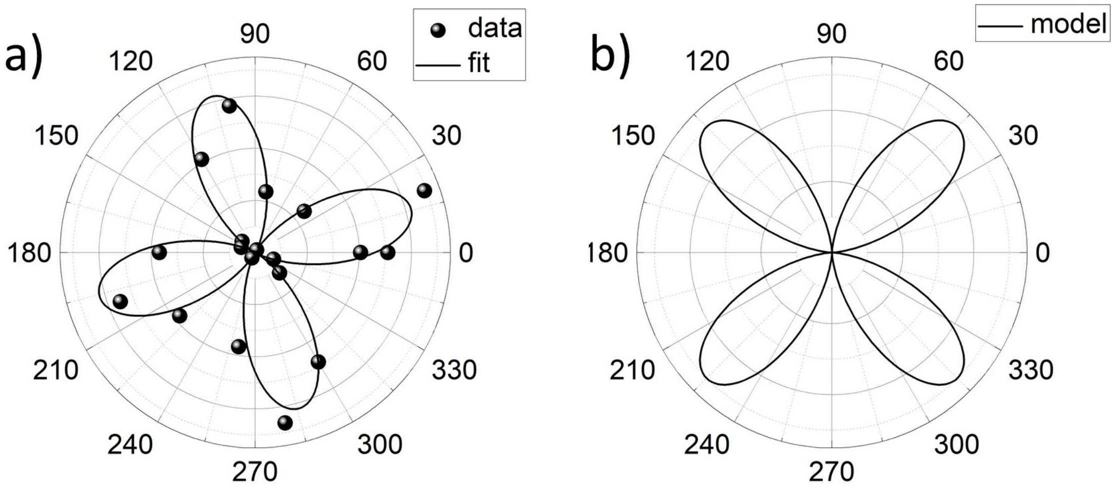

Figure S3: Angular intensity plot of silicon 520 cm^{-1} Raman mode in sample rotation configuration: a) experiment data with a fitted function, b) model.

Room temperature measurements

Raman spectra dependent on the scattered light polarization rotation obtained at room temperature are shown in Fig S4. As can be seen, some lines originating from ordered domains in the crystal lattice are visible. The most intense line at 241 cm^{-1} is already linearly polarized, which indicates that arranged parts of the structure exhibit polarization-dependent properties in the NCCDW phase.

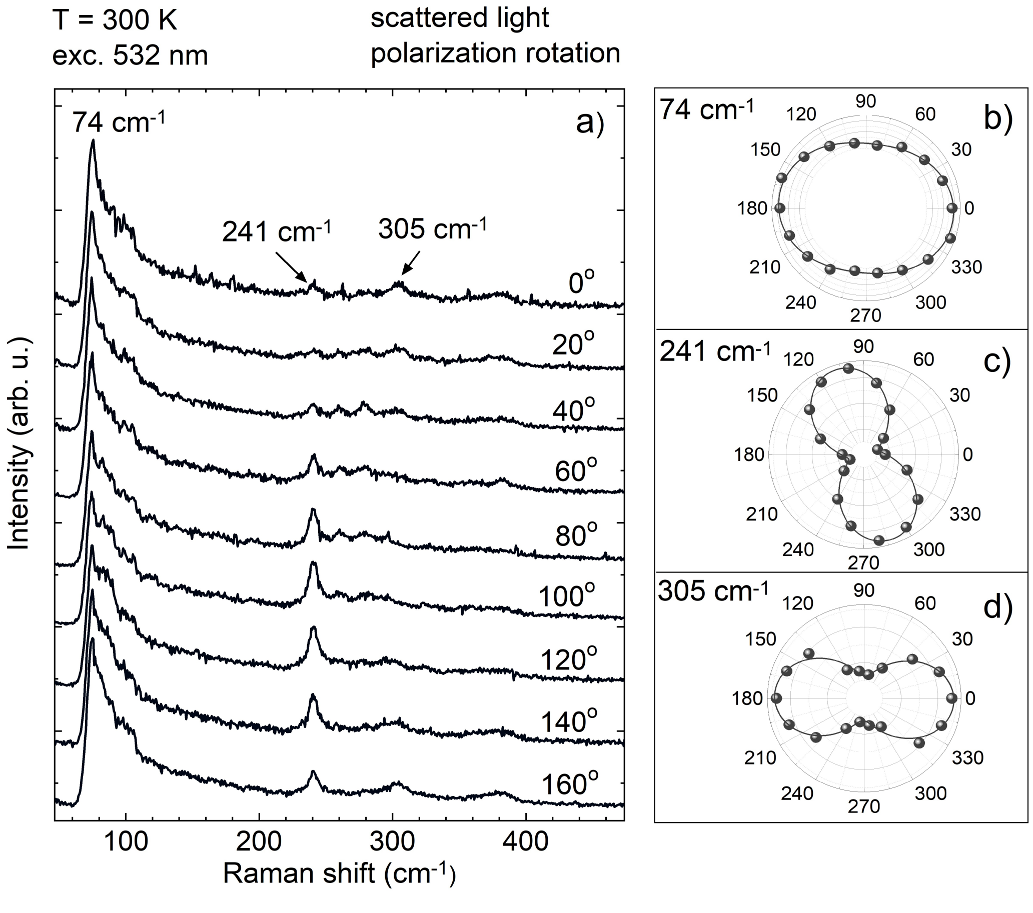

Figure S4: a) Room temperature bulk 1T-TaS_2 Raman scattering spectra measured in scattered light polarization rotation configuration under 532 nm wavelenght excitation. b) - d) Angular intensity plots of three most pronounced lines at 74, 241 and 305 cm^{-1} with the fitted function: $I = A + Bcos^2(\theta - \theta_{max})$. Low energy modes (below 72 cm^{-1}) are attenuated by the Raman filter.

Raman tensors and angular dependencies

Raman tensors of A_g and E_g modes for C_{3i} symmetry point group are given by [1]:

$$A_g = \begin{pmatrix} a & 0 & 0 \\ 0 & a & 0 \\ 0 & 0 & b \end{pmatrix} \tag{1}$$

$$E_g^1 = \begin{pmatrix} c & d & e \\ d & -c & f \\ e & f & 0 \end{pmatrix} \tag{2}$$

$$E_g^2 = \begin{pmatrix} d & -c & -f \\ -c & d & e \\ -f & e & 0 \end{pmatrix} \tag{3}$$

Based on the semi-classical model, the Raman mode intensity I can be written as [2]:

$$I = |{}^t e_s R e_i|^2, \tag{4}$$

where e_i and e_s stand for incident and scattered light electric field unit vector, respectively, and R is Raman tensor for given mode. Superscript t of e_s denotes the transformation from a column vector to a row vector.

In case of scattered light polarization dependent measurements, the incident light polarization angle is fixed, and we can write $e_i = (\cos\theta_0, \sin\theta_0, 0)$, where θ_0 is a constant angle between the incident light polarization direction and the crystal's axis. e_s depends on the angle set on the halfwave plate $\theta/2$, which is changed during the experiment, and can be written as: $e_s = (\cos(\theta_0{+}\theta), \sin(\theta_0{+}\theta), 0)$. Applying Raman tensor for each Raman mode gives:

$$I_{A_g} = a|\cos\theta|^2, \tag{5}$$

$$I_{E_g^1} = |\cos(\theta+\theta_0)(c\cos\theta_0 + d\sin\theta_0) + \sin(\theta+\theta_0)(d\cos\theta_0 – c\sin\theta_0)|^2, \tag{6}$$

$$I_{E_g^2} = |\cos(\theta+\theta_0)(d\cos\theta_0 – c\sin\theta_0) – \sin(\theta+\theta_0)(c\cos\theta_0 + d\sin\theta_0)|^2. \tag{7}$$

Modelled angular dependencies for scattered light polarization rotation are shown in Fig S5.

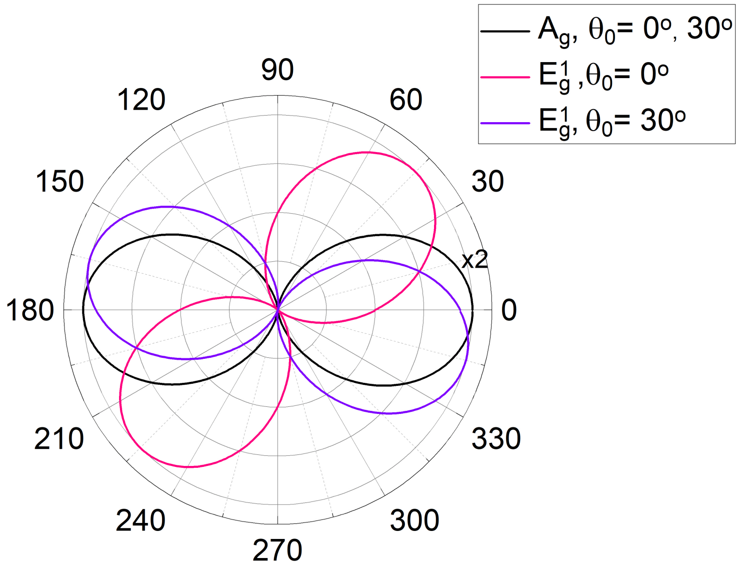

Figure S5: Modelled A_g and E_g Raman modes angular intensity plots dependent on the scattered light polarization rotation for the sample rotated by 30° for crystal with C_{3i} point group. A_g modes stay the same, while E_g modes' main axis rotates significantly (by 60°).

In sample rotation experiment θ_0 is changed during the measurements thus e_i and e_s can be written as: e_i=e_s=$(\cos\theta_0, \sin\theta_0, 0)$. This results in Raman modes intensities (dependent on the θ_0 value):

$$I_{A_g} = |a|^2, \tag{8}$$

$$I_{E_g^1} = |\cos\theta_0(c\cos\theta_0 + d\sin\theta_0) + \sin\theta_0(d\cos\theta_0 - c\sin\theta_0)|^2, \tag{9}$$

$$I_{E_g^2} = |\cos\theta_0(d\cos\theta_0 - c\sin\theta_0) - \sin\theta_0(c\cos\theta_0 + d\sin\theta_0)|^2. \tag{10}$$

Modelled angular dependencies for sample rotation case are shown in Fig. S6.

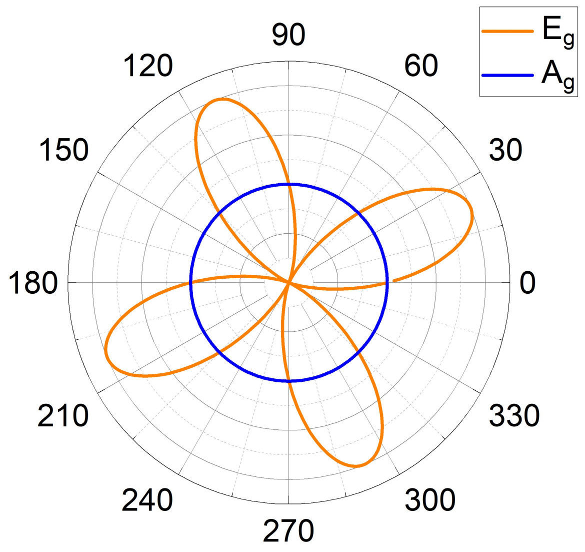

Figure S6: Angular intensity plot of A_g and E_g modes for crystal with C_{3i} symmetry point group in sample rotation configuration.

Fig. S7 shows a relation between the polarization degree of the Raman modes and their main axis rotation induced by a change of the excitation energy. As can be seen, only weakly polarized lines change their polarization direction. This finding suggests that weakly polarized lines may in fact consist of two (or more) components that can not be resolved by our setup, which can be enhanced by different lasers.

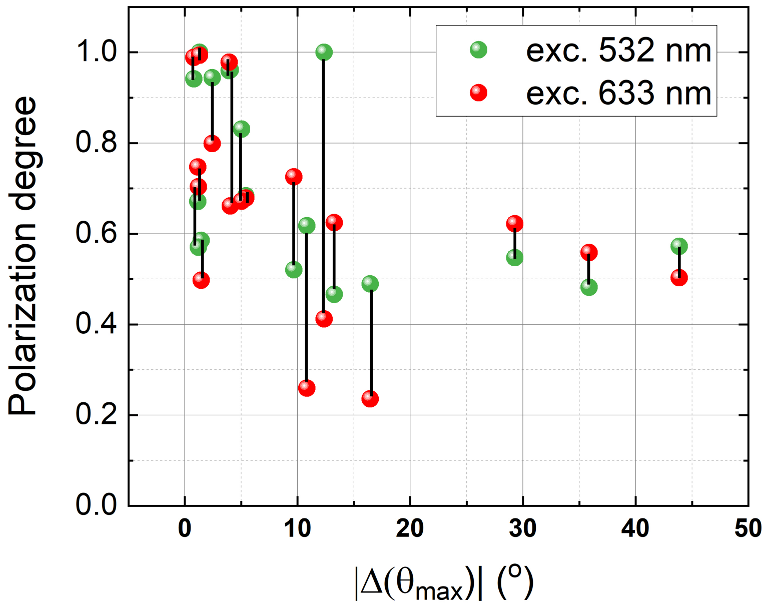

Figure S7: Dependence of the polarization degree of Raman modes as a function of polarization main axis rotation ($| \Delta(\Theta_{max} |$) induced by change of excitation line from 532 nm to 633 nm.

Measurements in circularly polarized light

Figs S8-S9 show Raman spectra of bulk 1T-TaS_2 obtained in circularly polarized light in different configurations.

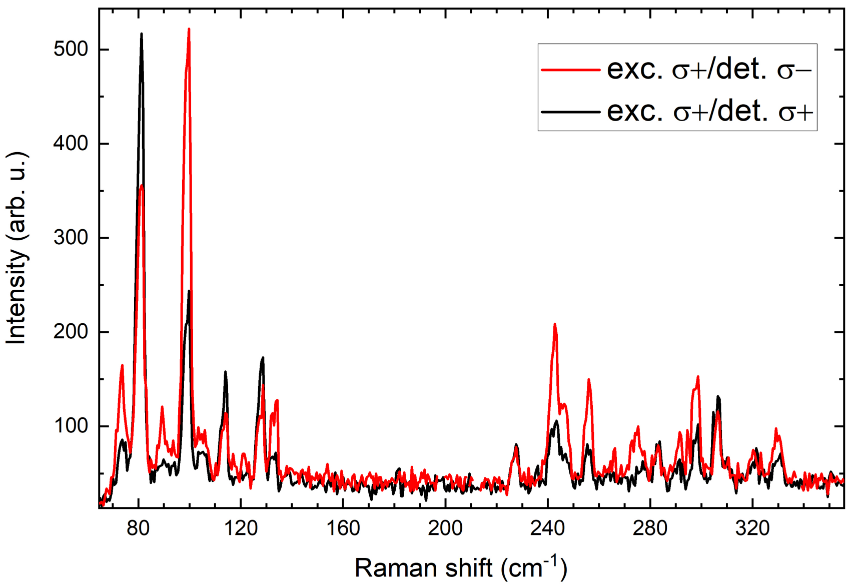

Figure S8: Bulk 1T-TaS_2 Raman spectra measured in circularly polarized light: σ+/σ- (red line) and σ+/σ+ (black line).

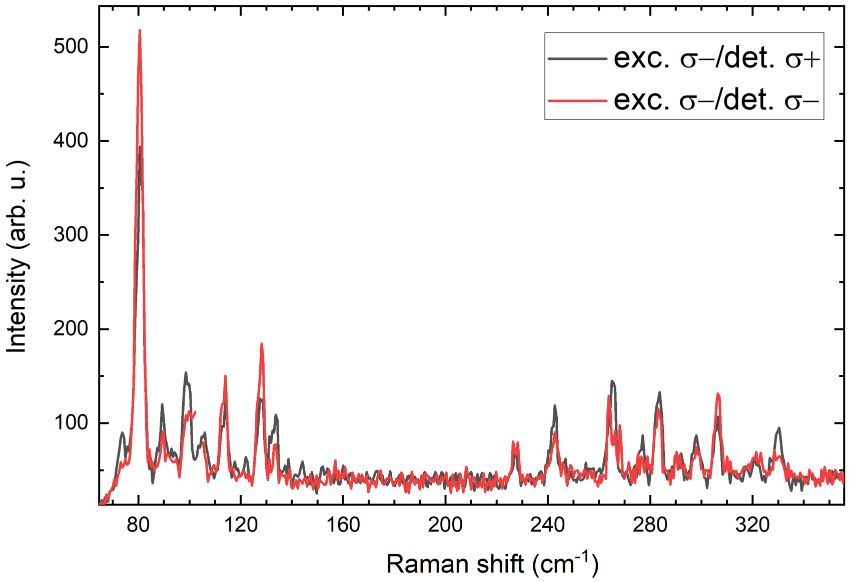

Figure S9: Bulk 1T-TaS_2 Raman spectra measured in circularly polarized light: σ-/σ+ (red line) and σ-/σ- (black line).

References

[1] R.C. Powell. *Symmetry, Group Theory, and the Physical Properties of Crystals.* Springer, 2010.

[2] R. Saito et al. "Raman spectroscopy of transition metal dichalcogenides". In: *Journal of Physics: Condensed Matter* 28 (2016), p. 353002. DOI: 10.1088/0953-8984/28/35/353002.
